# Lymphoma in HIV patients: Varied presentations

**DOI:** 10.4103/0971-5851.68854

**Published:** 2010

**Authors:** Dattatray G. Saple, Ira Shah, Amar U. Surjushe, Anuradha Murthy, Priya Chudgar, Prashant D. Gote

**Affiliations:** *Department of Dermatology, Venereology and Leprosy, Gokuldas Tejpal Hospital, Grant Medical College, Mumbai, India*; 1 *Department of Pediatric HIV, B. J. Wadia Hospital for Children, Mumbai, India*; 2 *Department of Pathology, B. J. Wadia Hospital for Children, Mumbai, India*; 3 *Department of Radiology, Nivaran CT Scan Centre, Mumbai, India*

**Keywords:** *HIV*, *lymphoma*, *non-hodgkins lymphoma*

## Abstract

Although lymphomas have been reported in patients with acquired immunodeficiency syndrome, it has rarely been reported from the Indian subcontinent. We present three human immunodeficiency virus-infected patients (two adults and one child) who had non-Hodgkin’s lymphoma - plasmablastic variety, Hodgkin’s lymphoma - nodular sclerosis type II and B cell lymphoma, respectively.

## INTRODUCTION

Acquired immunodeficiency syndrome (AIDS)-associated lymphomas are the second most common malignancies next to Kaposi’s sarcoma. The incidence of non-Hodgkin’s lymphoma (NHL) has increased with the AIDS epidemic, accounting for 2%–3% of newly diagnosed AIDS cases.[1] Human immunodeficiency virus (HIV)-associated lymphomas include (1) high-grade B-cell lymphomas: Burkitt lymphoma, diffuse large B-cell lymphoma with centroblastic features and with immunoblastic features and (2) unusual lymphomas, “primary effusion lymphoma” and “plasmablastic lymphoma” of the oral cavity. We present three cases of lymphoma in HIV patients with varied manifestations.

## CASE REPORTS

### Case 1

**Figure 1:**
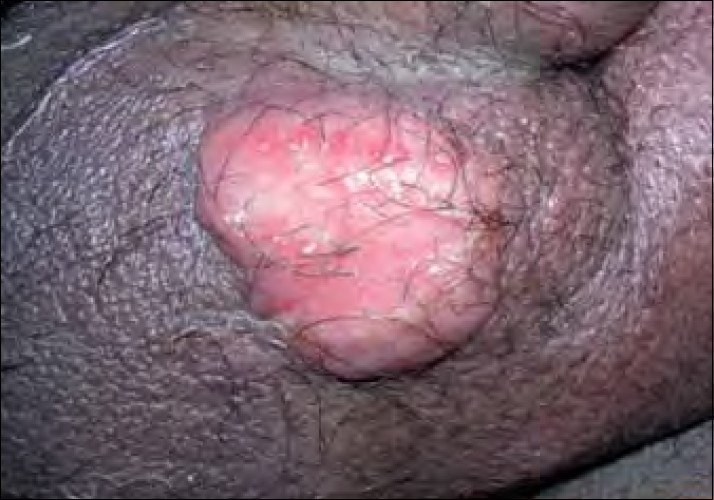
Patient 1: Sprouting erosive growth over the scrotum

A 35-year-old male detected seropositive for HIV-1 diagnosed recently not on antiretroviral therapy (ART) presented with painful swelling over the genital, inguinal and periumbilical regions with distension of the abdomen since 1 month. There was a sudden onset of genital swelling followed by redness and severe throbbing pain. He had high-grade intermittent fever with weakness, loss of weight and appetite. On examination, he had pallor and bilateral inguinal lymphadenopathy. Cutaneous examination showed erythematous annular tender indurated plaque with well-defined irregular margins of size 7 cm×8 cm around the umbilicus. A diffuse erythematous, indurated, tender swelling of size 6 cm×10 cm was present over the penis and scrotum with sprouting erosive growth over the scrotum [Figure 1]. A differential diagnosis of cellulitis, cutaneous tuberculosis, lymphoma, Kaposi’s sarcoma and histoplasmosis was considered.

On investigation, the patient had hemoglobin of 11.5 g%, total leukocyte count of 2,500/mm^3^, absolute lymphocyte count of 475 cell/mm^3^, adequate platelets count and erythocyte sedimentation rate (ESR) of 37 mm at the end of 1 h. Serum electrolytes, urine, stool, liver and renal function tests were normal. Venereal Disease Research Laboratory Research (VDRL), Hepatitis B surface antigen (HbsAg), Mantoux test, pus for acid fast bacillus (AFB) and sputum for AFB were negative. X-ray chest showed right-sided pleural effusion. Sonography of the abdomen and pelvis showed thickened anterior abdominal wall, retroperitoneal fibrosis, liver parenchyma disease and bilateral vaginal hydrocele. Ultrasonography chest showed right-sided pleural effusion with moderate pericardial effusion on echocardiography. Computerized tomography (CT) of the abdomen showed extensive abdominal subcutaneous fat with hypodensity in internal oblique to the left pararenal space. The CD4 count was 135/mm^3^. Skin biopsy showed dense infiltrate seen in the dermis with larger cells with formation of slits. Higher magnification showed characteristic splindeloid cells with hyperchromatic nuclei and scant cytoplasm. Tumor cells expressed epithelial membrance antigen (EMA), with possible kappa light-chain restriction. Thus, the final diagnosis of NHL, plasmablastic variety was made.

The patient was started on low-dose Cyclophosphamide, hydroxydoxorubicin (Oncovin), vincristine, prednisolone (CHOP) therapy. There was a significant reduction in eryrthema, induration and size of the lesion after two cycles. The patient took discharge against medical advice and succumbed after 1 month at home.

### Case 2

A 40-year-old male, seropositive for HIV-1, diagnosed 2 months back, presented with cough with expectoration, breathlessness on exertion, bilateral edema feet and intermittent fever since 2 months. He had history of painless swelling over the neck with loss of weight and appetite since 1 month. He was started on antituberculous therapy (ATT) since 2 months for pulmonary tuberculosis. On examination, he had diffuse swelling over the posterior cervical region, 4 cm×3 cm, nontender and firm to hard in consistency. On systemic examination, he had muffled heart sounds, bilateral basal crepts and splenomegaly. He had hemoglobin of 6.3 g%, total leukocyte count 8,300/mm^3^, adequate platelet count, absolute lymphocyte count 747 cells and ESR 32 mm at the end of 1 h. Renal and liver function tests and electrolytes were normal. Mantoux test, sputum for AFB, HbsAg and VDRL were negative. His CD4 count was 166 cells/mm^3^. The chest radiograph showed superior mediastinal widening and cardiomegaly. Sonography of the chest and abdomen showed minimal pericardial effusion and splenomegaly with extensive periportal lymphadenopathy. He had dilatation of all four chambers of the heart [left ventricular ejection fraction (LVEF), 72%] on echocardiography. CT of the chest and abdomen showed extensive mediastinal and abdominal lymphadenopathy. Fine needle aspiration revealed multinucleated Reed Steinberg (RS)-like large cells with abundant pale cytoplasm, with lobular nuclei and coarse chromatin. Lymph node biopsy showed RS cells and dense fibrous tissue. RS cells expressed CD 30 and were negative for LCA, CD20, CD15 and EMA. A final diagnosis of Hodgkin’s lymphoma, nodular sclerosis type-II (Ann Arbor stage IV-A) was made. The patient received six cycles of low-dose and two cycles of standard-dose CHOP therapy with ART, consisting of Stavudine (d4T)+Lamivudine (3TC)+Nevirapine (NVP) along with Digoxin. After eight cycles, the patient was asymptomatic, with reduction in neck swelling and weight gain. His CD4 improved from 166 to 247 cells/mm^3^, with reduction in size of the mediastinal and abdominal lymph nodes on sonography and CT scan.

### Case 3

**Figure 2:**
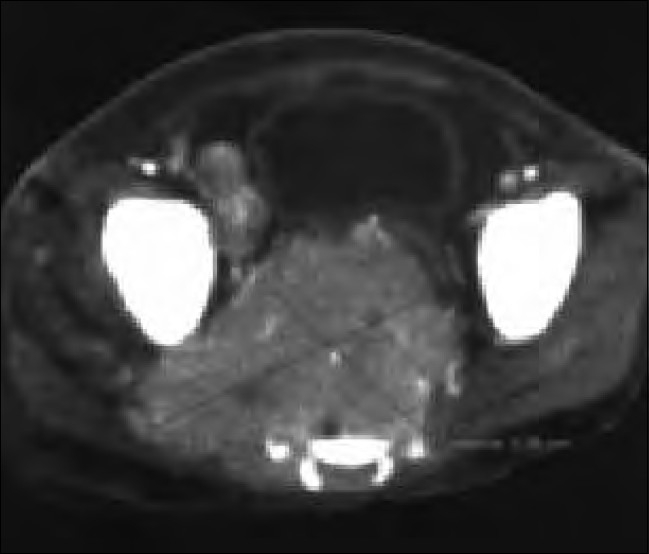
Patient 3: Computerized tomography (CT) of the abdomen and pelvis. Contrast-enhanced axial CT image through the lower pelvis. Heterogeneously enhancing soft tissue mass destroying the sacrum. It displaces the urinary bladder anteriorly and also extends into the right side. Another large lymphnode mass is also noted adjacent to the iliac vessels

A 5 1/2-year-old HIV-infected boy presented with recurrent fever and cough since 2 years, recurrent diarrhea and vomiting since 6 months and generalized pruritis and abdominal pain since 15 days. He had taken ATT 2 years ago for 6 months. His father was a prisoner and was diagnosed to be HIV-infected 2 years back. His mother was also diagnosed to be HIV infected a year back. Thus, the child seemed to have acquired the HIV infection vertically. He was immunized till date. On examination, he was malnourished (weight=10 kg, <5^th^ centile and height=96 cm, <5^th^ centile). He had pallor, multiple mobile nontender cervical, axillary and inguinal lymphnodes with oral thrush. On systemic examination, he had mild splenomegaly. Other systems were normal. His investigations showed hemoglobin of 11.4 g/dl, white cell count of 7,200/mm^3^ and ESR of 62 mm at end of 1 h. X-ray chest showed bilateral hilar congestion. Mantoux test was negative. Liver function and renal function tests were normal. His enzyme-linked immunosorbent assay for HIV was repeated, which was positive. A baseline CD4 count of 161 cells/mm^3^ was detected, with a CD4 percentage of 7.98%, suggestive of severe immunosuppression. He was started on three drugs; ART consisting of Stavudine, Lamivudine and Efavirenz. He had a weight gain of 2 kg within 1 month of starting ART and his lymphadenopathy regressed. However, his cough and pruritis persisted. A repeat X-ray chest showed a right-sided lower zone pneumonia. A Mantoux test performed at this time was negative. He was treated with antibiotics but the pneumonia did not subside, and thus he was started on ATT to which the pneumonia responded. Three months after start of ATT, he developed left cervical matted rubbery lymphadenopathy (4 cm×4 cm) that did not subside with antibiotics. Fine needle aspiration of the cervical adenopathy was carried out, which showed caseous granulomas. Drug compliance with ATT was confirmed. A wedge biopsy of the node was carried out, which again confirmed tuberculosis, and then the child was started on six-drug ATT. Subsequently, within 1 week, he developed severe sepsis with intestinal obstruction and was hospitalized. CT of the abdomen and pelvis showed multiple splenic lesions, a large liver lesion, encasing retroperitoneal lymphnodes, large right pelvic lymphnodes and a large right inguinal lymphnode with a large presacral mass (6.8 cm×5.08 cm) with intrasacral extension into the spinal cord suggestive of lymphoma [Figure 2]. However, before any intervention could be started, the child expired the following day. A postmortem lymph node biopsy was performed, which showed round cell tumor that was found to be a B cell lymphoma on immunophenotyping.

## DISCUSSION

HIV infection significantly increases the risk for the development of lymphoma. NHL is present in about 3% of the HIV-positive people at the time of their diagnosis of HIV. Twenty percent of HIV-positive patients develop NHL.[1] Risk factors for the development of NHL in HIV include a low CD4 T-cell count, high HIV viral load, increased age and male gender.[2] In the study described by Agarwal et al., of 35 cases, seven cases were of Hodgkin disease, four of plasmacytoma and 24 cases were of NHL (three Burkitt’s lymphoma, four diffuse large B-cell lymphoma of centroblastic type, 10 immunoblastic type, four high-grade B-cell lymphoma [unspecified] and the remaining were other subtypes).[3] In our patients, three different varieties of lymphoma were found, namely NHL (plasmablastic variety), Hodgkin’s lymphoma, nodular sclerosis type-II and B cell lymphoma, respectively. The diagnosis of AIDS precedes the onset of NHL in approximately 57% of the patients, but in 30%, the diagnosis of AIDS is made at the time of the diagnosis of NHL and HIV positivity.[4] All our patients were diagnosed to be HIV infected prior to their diagnosis of lymphoma and all were already having AIDS at the time of presentation.

Plasmablastic lymphoma, originally described in 1997 in a series of 16 patients,[5] is highly associated with advanced stages, and accounts for 2.6% of all HIV-related NHL. It is found with Ebstein barr virus (EBV) (15%) and Human Herpes virus 8 (HHV8) (38%) infection. Average age of onset is 33 years, much younger than would be expected for HIV-negative individuals, and commonly involves jaws, oral cavity, stomach, anorectum, nasal-paranasal areas and lungs.[6,7] Our patient presented with cutaneous lesions, which appears to be rare. The tumors are characterized by immunoblastic morphology and plasma cell phenotype. Markers are positive mainly for LCA, CD79a, VS38C and CD138. In the pre-HAART era, prognosis of plasmablastic lymphoma was poor, with a median survival of about 5.5 months, although prognosis may have improved since the advent of HAART.

HIV-associated Hodgkin lymphoma, although not included in the CDC definition of AIDS, has been linked to HIV infection, with a relative risk of 11.5 in one study and predominance of two unfavorable subtypes: lymphocyte depleted and mixed cellularity.[8] HIV-associated Hodgkin’s lymphoma presents in an aggressive fashion, often with extranodal or bone marrow involvement.[9,10] A distinctive feature of HIV-associated Hodgkin’s lymphoma is the lower frequency of mediastinal adenopathy compared with non-HIV-associated Hodgkin’s lymphoma. In our case, the patient had extensive mediastinal adenopathy, which responded well with treatment.

AIDS-related lymphomas behave differently in the clinical setting and should be suspected in any patient with HIV who has a sudden increase in size of the lymphnode or presents with central nervous system (CNS) manifestations. Major differences from non-HIV patients are that these tumors have more aggressive clinical course, widespread involvement, are less responsive to chemotherapy and frequent relapse is seen. Chemotherapy consists of a much-reduced course of the standard chemotherapy regimens, half the dose of each component drug and for only four, rather than the usual 10, monthly treatments.[11] Because chemotherapeutic agents are immunosuppressants, treatment of the malignancy increases the risk of opportunistic infections, thereby requiring prophylaxis. HAART appears to be a major positive prognostic factor for patients with AIDS.[12] Among ART, Zidovudine is relatively contraindicated because of its marrow-suppressive effect. Protease inhibitors, mainly ritonavir, have the most significant effect on the cytochrome p450 system and hence increase the toxicities of chemotherapy agents. In the HIV-infected child in our series, the child was already on ART for 4 months. Most of the patients who have developed malignancy are the ones who were not on ART.

Prognosis of patients with AIDS-related lymphoma has been associated with extent of disease, extranodal involvement and bone marrow involvement, CD4 lymphocyte count, performance status and prior AIDS diagnosis. Median survival time ranges from 8 to 20 months, which is much poorer than the survival expected in patients with non-HIV-associated Hodgkin’s lymphoma.
